# A Patient- and Function-Centric Model/Approach for Hypoglycemia Management in Older Adults

**DOI:** 10.3390/geriatrics11050118

**Published:** 2026-09-03

**Authors:** Kannayiram Alagiakrishnan, Laurie Mereu, Gulelala Rahim, Mahua Ghosh, Albert Vu

**Affiliations:** 1 Division of Geriatric Medicine, University of Alberta, Edmonton, AB T6G 2B7, Canada; 2 Division of Endocrinology, University of Alberta, Edmonton, AB T6G 2B7, Canada; laurie.mereu@ualberta.ca (L.M.); mahua@ualberta.ca (M.G.); avu1@ualberta.ca (A.V.); 3 Department of Medicine, University of Alberta, Edmonton, AB T6G 2B7, Canada; gulelala@ualberta.ca

**Keywords:** diabetes, hypoglycemia unawareness, fear of hypoglycemia, hypoglycemia distress, function-centric approach, continuous glucose monitoring, intranasal glucagon

## Abstract

Diabetes-related hypoglycemia contributes substantially to increased morbidity, mortality, health-care utilization, and reduced quality of life. Older adults with diabetes represent a heterogeneous group of patients who need individualized blood glucose targets to avoid hypoglycemia. Since the elderly present with varying degrees of functional and cognitive status and individualized health needs, their management varies among individuals. Complicating this, the hypoglycemia risk in older adults treated with insulin also varies due to aging, renal dysfunction, cognitive impairment, and other comorbidities. The challenge for healthcare providers is in considering all aspects of care in order to avoid hypoglycemia in elderly individuals. In this review, we introduce a Patient and Function Centric Approach to the assessment and management of hypoglycemia in older adults. This holistic framework extends beyond blood glucose values to systematically evaluate the other domains of patients’ health that influence hypoglycemia risk, including biochemical, medication, timing, autonomic, cognitive, mood, renal, pancreatic, hepatic, social, and physical function. The management of hypoglycemia in older adults should also include strategies to address both fear of hypoglycemia and hypoglycemia unawareness. In addition to physical limitations, the psychosocial barriers to self-care in older individuals with hypoglycemia are also of paramount importance. Using tools that measure diabetes burden, diabetes distress, and fear of hypoglycemia provides valuable insights into patient wellbeing. The use of newer anti-hyperglycemic medications, sensor-augmented insulin pump therapy, intranasal glucagon, and continuous glucose monitoring (CGM) has significantly contributed to reduced hypoglycemia incidence in individuals with diabetes. Overall, successful management requires a collaborative approach that empowers patients, respects their individual needs and preferences and helps them face the challenges associated with diabetes management with confidence rather than fear or anxiety. Adopting a patient- and function-centric approach to hypoglycemia management allows clinicians to move beyond a one-size-fits-all model and adopt individualized glycemic targets that improve overall diabetes control and mental well-being.

## 1. Introduction

Hypoglycemia is one of the most serious adverse effects of diabetes management, defined as a plasma glucose concentration of <70 mg/dL (3.9 mmol/L) [1] In medicine where excellent glycemic control has proven to be the most effective strategy to reduce microvascular complications of diabetes, hypoglycemia remains the most daunting barrier in achieving this goal. Limited data on the incidence and prevalence of hypoglycemia are available, especially in older patients with type 2 diabetes mellitus (T2D). However, adverse events associated with hypoglycemia are frequently seen in clinical practice and well-documented in the literature.

The acute complications of hypoglycemia include susceptibility to falls, cognitive decline, increased vulnerability to seizures, loss of consciousness with the predominance of neurological rather than autonomic symptoms, and even death. Hypoglycemia may present with symptoms such as dizziness or visual disturbance, resulting in the possibility for misdiagnosis.

The HYPOTHESIS study showed that severe hypoglycemic events in elderly patients with diabetes were associated with frequent use of emergency medical services, high rates of hospitalization, and significant mortality [2]. A retrospective analysis of inpatient glycemic data from 16,935 older adults with type 2 diabetes revealed that those treated with insulin or sulfonylureas experienced the highest rates of hypoglycemia (3.5%), delirium (4.8%), cardiovascular complications (20.2%), and combined emergency department visits and hospitalizations (49%) [3]. However, in older adults, hypoglycemic events are common and recurrent, and likely to be under-recognized and under-reported both by patients and healthcare professionals.

A function-centric approach to hypoglycemia management is needed to shift focus from solely targeting blood glucose levels to improving the patient’s quality of life and overall health. It is a personalized and patient-centered approach, considering the interconnectedness of various physiological systems within the body as well as lifestyle factors and environment, and the underlying imbalances that may contribute to health issues [4].

In this review article, we discuss an integrative patient-centered approach to hypoglycemia management. This comprehensive framework moves beyond strict “glucocentric” (HbA1c-focused) strategies to a more holistic model that prioritizes individual needs, personalized control rather than strict targets, improving quality of life, safety, addressing emotional distress (like fear of lows), and using tools like CGM to monitor glucose while preserving function and well-being.

### 1.1. Methodology: Search Strategy

A literature search was performed using the electronic databases MEDLINE (1966–April 2026), EMBASE and SCOPUS (1965–April 2026), and DARE (1966–April 2026). The main search items were diabetes; hypoglycemia, hypoglycemia unawareness; fear of hypoglycemia; hypoglycemia distress; cognitive impairment, dementia, depression, anxiety, function-centric approach; continuous glucose monitoring; and intranasal glucagon. This article is a non-systematic, narrative review. Non-English articles were excluded.

### 1.2. Epidemiology

The frequency of hypoglycemia varies from 42 to 91 events per patient year for adults with type 1 diabetes mellitus (T1D) and from 20.3 to 44.4 events per patient year for adults with T2D. Data from population-based studies highlight how adults with type 1 diabetes face much higher rates of low blood sugar than adults with type 2 diabetes. There are no reported disparities in hypoglycemia incidence based on gender, but it is seen more frequently in patients with T1D compared to those with T2D [5]. This discrepancy is largely driven by the necessity of insulin use in T1D compared to patients with T2D, who may be treated with antihyperglycemic medications that are not associated with hypoglycemia.

A US population-based retrospective study of 279,937 patients showed that hypoglycemia-related hospital admissions exceeded admissions for hyperglycemia. Of the patients hospitalized for hypoglycemia, the incidence in octogenarians was double that of the other age groups [6,7].

The LIGHTNING study showed that the rates of severe hypoglycemia were approximately 50% lower with Glargine-300 than with Glargine-100 or IDet (insulin detemir) in insulin-naïve individuals, and 30% lower versus IDet in BI (basal insulin) switchers (all *p* < 0.05) [8].

### 1.3. Severity of Hypoglycemia

Hypoglycemic episodes are classified as severe when assistance is needed from another individual at the time of hypoglycemia, and non-severe when the episode is managed by the patient alone [9].

### 1.4. Hypoglycemia with Different Types of Diabetes

#### 1.4.1. Hypoglycemia in Type 1 Diabetes (T1D)

All patients with T1D are dependent on insulin therapy due to absolute insulin deficiency following the loss of functioning pancreatic beta cells. Hypoglycemia in elderly patients with T1D is more common due to the mismatch of insulin, carbohydrate intake and physical activity. The consequences in this population are often more profound than in younger patients, due to reduced functional reserve and a lack of brisk autonomic or counter-regulatory responses.

#### 1.4.2. Hypoglycemia in Type 2 Diabetes (T2D)

Elderly patients with T2D may require oral anti-hyperglycemic agents, insulin, or both to achieve glucose targets. When patients with advanced T2D become insulin-deficient, the frequency of severe hypoglycemia is similar to that among patients with T1D. In patients with T2D, treatment with insulin for 10 years is an important predictor of increased risk for hypoglycemia [10].

### 1.5. Patterns of Hypoglycemia

Different patterns of hypoglycemia can be seen in the elderly, including (a) fasting or early morning hypoglycemia, (b) postprandial hypoglycemia, and (c) nocturnal hypoglycemia.

Predictors and Potentiators of Hypoglycemia in Older Adults: Please see Table 1.

### 1.6. Clinical Presentation of Hypoglycemia

Typical hypoglycemia symptoms can be classified as either neurogenic (arising from sympathetic nervous system activation—sweating, tachycardia, palpitations, tremors, anxiety, nausea, vomiting) or neuroglycopenic (resulting from the brain’s lack of glucose). Atypical presentations can include confusion, unusual behavior (dementia-related symptoms), difficulty with routine tasks, loss of coordination, slurred speech, or blurry vision (Table 2). In older adults, it can present atypically as otherwise unexplained falls [12].

The most frequently observed symptoms in patients include sweating, dizziness, tachycardia, weakness, headache, tremors, blurred vision, and irritability (Table 2). In a recent cross-sectional study in rural areas of India, hypoglycemia with dizziness was reported as the most common symptom occurring in approximately 72.3% of patients [12]. A prospective study conducted by Al Khaldi YM et al. reported sweating (49%), palpitations (48%), blurred vision (31%), dizziness (9%), and headache (7%) as the most frequent symptoms experienced during hypoglycemic episodes in diabetic patients [13].

### 1.7. Complications of Hypoglycemia

Acute hypoglycemia poses many immediate safety risks in the elderly, including loss of consciousness, seizures, falls, and fall-related injuries such as fragility fractures and head trauma. Hypoglycemia in this patient population is associated with slower recovery times due to diminished physiological reserves, polypharmacy, and comorbidities such as renal dysfunction.

Additional serious complications associated with hypoglycemia include vision loss, neurocognitive decline, cerebrovascular disease, myocardial infarction, and cardiac arrhythmias in certain patients [13,14].As diabetes progresses, many patients develop impaired counter-regulatory mechanisms, making them more susceptible to the harmful effects of low blood glucose. Hypoglycemia activates the autonomic nervous system and triggers a surge in catecholamines, leading to increased heart rate and cardiac output. It also causes changes in cardiac electrophysiology due to catecholamine-induced hypokalemia and QT interval prolongation, as well as ST-segment depression. Additionally, hypoglycemia promotes a prothrombotic and pro-inflammatory state that can lead to endothelial dysfunction and atherosclerosis as well as increased CV morbidity and incidence of geriatric syndromes like polypharmacy, cognitive impairment /dysfunction [15,16,17,18]. These comorbidities increase the likelihood of severe hypoglycemia and associated mortality in this high-risk population.

The psychosocial effects of hypoglycemia are an under-recognized source of patient and caregiver distress. This can often be seen with recurrent hypoglycemia, which leads to medication non-adherence, disruption of work and associated financial stress, and fear of hypoglycemia [11,15].

### 1.8. Broader Social Impacts of Hypoglycemia

On a societal level, hypoglycemia also has an impact on public safety, as it contributes to workplace and motor vehicle accidents [16].

Hypoglycemia can have significant economic implications by placing a burden on the healthcare system and negatively impacting workplace productivity. A 2019 meta-analysis found that inpatient hypoglycemia was linked to longer hospital stays and higher in-hospital mortality [17] A recent analysis estimated that the direct medical cost of a hypoglycemic episode requiring medical intervention is nearly USD 1200, while productivity losses associated with severe hypoglycemia range from USD 160 to USD 580 [18]. On a larger scale, hypoglycemia resulted in an average loss of 58 workdays per 100 person-years for patients or family members in T1D and 19 lost workdays in T2D [19].

Hypoglycemia poses a significant barrier to optimal glycemic control, and its prevention should be a key principle in the management of diabetes among older adults. Minimizing hypoglycemic events by continuous glucose monitoring, modifying pharmacotherapy and health education may help to reduce the costs associated with hypoglycemia Complete dependence, defined as a BI (Barthel Index) score < 30, was also more common in patients with hypoglycemia (69.2% vs. 50%) [20].

### 1.9. Intensive Glycemic Control and the Risk of Hypoglycemia

Four landmark randomized controlled clinical trials that investigated patients with T2D (UKPDS; ADVANCE; ACCORD; and VADT) demonstrated that intensive glycemic control increased the risk of severe hypoglycemia [20,21,22]. Of these studies, the most significant is the ACCORD trial, which was discontinued prematurely due to excess all-cause mortality in the intensive treatment arm targeting a HbA1c below 6.0%. This coincided with, and was likely driven by, a three-fold increase in severe hypoglycemia in the intensive treatment group. Despite this, the aggressive HbA1c-lowering strategy did not show a reduction in cardiovascular events. The ACCORD findings demonstrated that the risks of aggressive glucose-lowering in older adults with diabetes exceed the possible benefits [23].

A 2022 meta-analysis of adults older than 60 years with T2D corroborated these findings with a larger sample size, highlighting that relaxed glycemic targets are safer and more appropriate than intensive control in older adults [24]. The same trend is seen with inpatients. A study in critically ill patients showed that those with hypoglycemia had a significantly higher hospital mortality rate compared to those without hypoglycemia (48.9% vs. 15.9%) [25]. However, an increase in intra-ICU mortality was not seen with hyperglycemia, providing further evidence against the need for aggressive glycemic control in hospitalized and acutely ill patients [26]. A national retrospective observational cohort study performed by Moser et al. showed that in addition to severity, the frequency of hypoglycemic episodes predicted premature death in T1D as well [27].

## 2. Function-Centric Approach to Hypoglycemia Assessment in Clinical Practice

**Figure 1:**
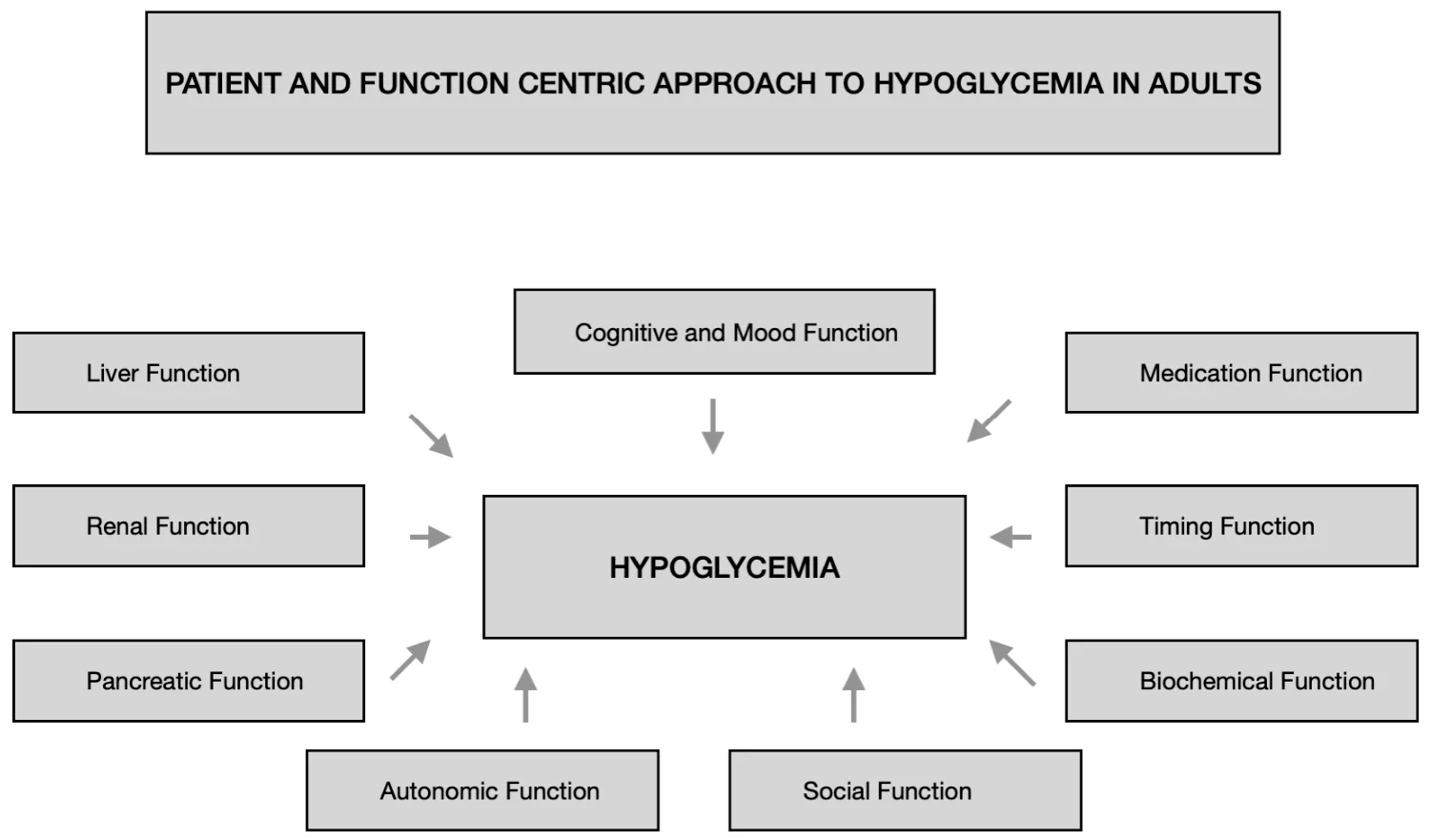
Patient- and function-centric approach to assessment of hypoglycemia. Different arrows show how hypoglycemic episodes compound the risk to different functions in older adults.

A function-centric approach focuses on identifying the root causes of disease rather than just managing symptoms (Figure 1). By analyzing the interactions between genetics, environment, and lifestyle, personalized treatment plans can be developed.

### 2.1. Biochemical Function

#### 2.1.1. Point-of-Care (POC) Capillary Glucose 

Point-of-care (POC) capillary glucose testing before meals and at bedtime is commonly used in hospital settings for monitoring insulin therapy in older adults. However, in critically ill patients with impaired perfusion, these POC glucose readings may not be accurate. In such cases, continuous glucose monitoring (CGM) may provide more comprehensive data, including identifying trends and detecting asymptomatic hypoglycemia that may not be revealed by traditional POC testing. CGM metrics also create an important feedback loop that enhances treatment adherence, improves patient experience, and supports informed treatment decision-making between patients and their healthcare team [28].

A recent randomized controlled trial demonstrated that CGM combined with remote virtual diabetes educator support led to better HbA1c control and improved patient satisfaction compared to usual care in adults with T2D not on insulin therapy. Although this study did not focus exclusively on older adults, its findings support the broader use of CGM as a proactive tool to prevent hypoglycemia [29]. Another qualitative study exploring perceptions of CGM among older adults with insulin-treated T2D found that it was viewed as valuable for reducing hypoglycemia risk, guiding behavior, and supporting informed decision-making. The findings highlight CGM’s potential to enhance hypoglycemia prevention and improve overall diabetes management in this population [30]. In CGM trials among older adults with T1D or T2D (MOBILE, DIAMOND), CGM use led to increased time spent in the target glucose range and reduced hyperglycemia without increasing time spent in hypoglycemia [31,32].

#### 2.1.2. Continuous Glucose Monitoring Metrics

Time in range (TIR) refers to the percentage of time glucose levels are within 70–180 mg/dL (3.9–10 mmol/L). Time below range (TBR) represents the time spent below 70 mg/dL (<3.9 mmol/L). The recommended clinical target for TIR is greater than 70%, and for TBR, it is less than 1% [33]. A randomized clinical trial by Spanakis et al. showed that inpatient use of CGM to guide insulin therapy significantly reduced recurrent hypoglycemic events and improved overall blood glucose control in hospitalized patients with diabetes [34]. The advent of continuous glucose monitoring (CGM), continuous subcutaneous insulin infusion, and advanced machine learning-based glucose predictive models has paved the way for the development of automated insulin delivery systems (AIDs) [35]. These systems allow communication between CGM devices and insulin pumps utilizing algorithms to automatically adjust insulin delivery based on CGM data [36].

In summary, clinicians should consider CGM to detect recurrent hypoglycemia in older adults with diabetes, especially those treated with insulin. It provides more accurate and comprehensive glucose data than POC testing. While evidence shows CGM improves outcomes in this population, research remains limited in older adults with sarcopenia and reduced subcutaneous fat. Given that sensor performance can be affected by tissue composition, further research is needed to assess CGM usability in this vulnerable subgroup.

### 2.2. Medication Function

In older adults with diabetes, medication-induced hypoglycemia is most commonly caused by insulin, sulfonylureas (SUs), and meglitinides. SUs carry a high risk due to their prolonged duration of action, while meglitinides pose a lower risk due to their short-acting nature but still require caution if meals are delayed. Similarly, insulin-induced hypoglycemia often results from mismatched timing or mismatched dosing relative to food intake [37]. Clinicians should be especially cautious when prescribing high-risk medications to older adults, considering the variables like oral intake, increased frailty, and associated comorbidities. Newer anti-hyperglycemic agents like SGLT2 inhibitors, GLP-1 receptor agonists, and DPP-4 inhibitors pose a lower risk of hypoglycemia and hence should be preferred for use in older adults.

#### 2.2.1. Sulfonylurea-Induced Hypoglycemia

First-generation sulfonylureas (SUs), including chlorpropamide and glibenclamide, carry a high risk of hypoglycemia due to their prolonged half-life, especially in elderly patients. Consequently, they are no longer commonly used. Second-generation sulfonylureas, such as glyburide and gliclazide, have been linked to rapid and prolonged hypoglycemic episodes. To mitigate this risk, elderly patients should be initiated on low doses with gradual titration, or these agents should be avoided altogether in favor of alternatives with a low hypoglycemia risk. Physicians should also be vigilant about potential drug–drug interactions, as SUs can interact with medications like salicylates, sulfonamides, and allopurinol, which may enhance SUs’ effects by displacing them from plasma proteins or reducing the urinary excretion of SUs and their metabolites [38].

#### 2.2.2. Meglitinide-Induced Hypoglycemia

Meglitinides are ultra-short-acting agents primarily used to control postprandial glucose levels. However, in elderly patients’ hypoglycemia may occur more frequently than in younger individuals due to delayed gastric emptying, slow food intake, or both. Meglitinides, however, are associated with a lower risk of hypoglycemia compared to sulfonylureas when a meal is missed, making them a more favorable option in some older adults [39].

#### 2.2.3. Insulin-Induced Hypoglycemia

Hypoglycemia remains a major concern associated with insulin therapy despite it being the treatment of choice in T1D as well as in T2D when non-insulin antihyperglycemic therapy is insufficient. In the Diabetes Control and Complications Trial (DCCT), intensive insulin treatment was found to increase the risk of severe hypoglycemia, particularly when insulin dosing was not well-aligned with food intake [40].

Modern insulin formulations have been shown to lower the risk of hypoglycemia. A meta-analysis found that treatment with insulin lispro resulted in significantly fewer severe hypoglycemic episodes compared with regular insulin (4.4% vs. 31% of patients; *p* = 0.024). Insulin glargine provides a smoother and more prolonged time action profile, lasting up to 24 h with a less pronounced peak, and studies have demonstrated that it leads to fewer hypoglycemic events than NPH insulin. The SWITCH 2 randomized clinical trial compared insulin degludec (IDeg), an ultra-long-acting insulin with a duration of up to 42 h with insulin glargine U100 (IGlar) in adults with type 2 diabetes at high risk for hypoglycemia. During the 32-week maintenance phase, IDeg was associated with a 22% reduction in overall symptomatic hypoglycemia and a 42% reduction in nocturnal hypoglycemia relative to IGlar. Although the difference in severe hypoglycemia was not statistically significant, IDeg showed a lower incidence. Overall, IDeg provided comparable glycemic control with fewer hypoglycemic events, making it a favorable option for patients prone to hypoglycemia [41].

#### 2.2.4. Other-Medication-Induced Hypoglycemia

ACE inhibitors have been associated with an increased risk of hypoglycemia due to their ability to enhance insulin sensitivity. In contrast, nonselective beta-blockers can mask the autonomic symptoms of hypoglycemia by blunting the sympathetic response. In the UKPDS study, hypoglycemic episodes occurred in 6.5% of patients treated with ACE inhibitors compared to 4.4% of those receiving atenolol. Another study reported that ACE inhibitors were linked to a nearly threefold increase in the risk of hypoglycemia in diabetic patients, attributed to their insulin-sensitizing effects (odds ratio [OR] 2.8; 95% CI, 1.4–5.7) [42].

Other commonly used medications, including quinolones, sulfa drugs, anti-malarial quinine, lithium, SSRIs like sertraline, and angiotensin receptor antagonists, can also cause hypoglycemia [43]. However a recent study showed a lower incidence of hypoglycemia with ARBs when compared to ACE inhibitors [44,45].

### 2.3. Timing Function

#### 2.3.1. Timing of Hypoglycemia

A retrospective observational study conducted by Ruan et al. on 17,658 diabetic patients examined the distribution of hypoglycemic events. The study showed an increased frequency of hypoglycemia occurring approximately 3 h after lunch and dinner, with the highest number of episodes recorded between 1 a.m. and 5 a.m. [46]. Similarly, another study conducted by Kant et al. on circadian patterns of hypoglycemia showed a pattern of hypoglycemic episodes between 12 a.m. and 6 a.m. in around 55% patients [47]. Cun-Mei Yang et al. observed that most severe hypoglycemic episodes occurred during the midnight to 2 a.m. period [48]. Therefore, clinicians should be mindful of the circadian pattern of hypoglycemia in adults, enabling them to adjust and titrate insulin doses appropriately to minimize the risk of nocturnal and severe hypoglycemic events.

#### 2.3.2. Nocturnal Hypoglycemia

Older adults are particularly at risk of nocturnal hypoglycemia for a number of reasons.

For decades, patients on insulin have been counselled to self-monitor glucose by using POC blood glucose meters (BGMs). Traditionally, testing is performed at discrete time points throughout the day, such as before meals, after meals, and at bedtime. However, compliance with POC BGM monitoring may be suboptimal for many reasons, including cost, skin irritation, and alarm fatigue. Monitoring may also be particularly challenging for older adults with visual impairment, reduced manual dexterity, apathy or cognitive impairment. This often leads to less frequent testing and therefore reduced recognition of hypoglycemia.

Adult patients taking short-acting or intermediate-acting analogues such as regular insulin or twice-daily isophane insulin are at increased risk of nocturnal hypoglycemia due to their inherent peaks in glucose-lowering activity. Patients may not recognize overnight lows due to blunted adrenergic symptoms while sleeping, and clinicians may similarly lack awareness of these episodes due to a lack of overnight glucose records. As shown in the HYPOAGE observational study, which examined patients older than 75 years with insulin-treated diabetes who used CGM, 65% of participants experienced nocturnal hypoglycemia lasting more than 15 min between midnight and 6:00 a.m. [49]. Nocturnal hypoglycemia is rarely captured by self-monitored POC glucose tests because many of these episodes do not wake the person who is affected. Nocturnal hypoglycemia management involves a combination of immediate standard hypoglycemia treatment, medication adjustment, technology usage like CGM, and dietary modifications. In particular, CGM is a powerful tool to detect nocturnal hypoglycemia and to personalize patient management, especially for those with cognitive impairment.

- Treatment strategies for nocturnal hypoglycemia focus on acute treatment, identification of underlying causes, prevention, and patient education. Immediate management involves administration of 15–20 g of fast-acting carbohydrate, followed by blood glucose reassessment after 15 min. Once euglycemic, a longer-acting carbohydrate (e.g., toast or crackers), ideally with protein, should be given to prevent recurrence. In cases of severe hypoglycemia or loss of consciousness, glucagon (intramuscular, subcutaneous, or intranasal) should be administered promptly, and urgent medical care sought.
- A critical step in management is identifying precipitating factors, which commonly include excess basal insulin, long-acting sulfonylureas, evening or bedtime short-acting insulin, missed or inadequate bedtime snacks, evening alcohol intake, and increased daytime physical activity.
- Medication optimization is central to prevention. This may include reducing basal insulin doses, particularly when hypoglycemia occurs between 2–4 a.m., and switching to longer-acting insulin analogues such as glargine U300 or insulin degludec. Use low-hypoglycemia-risk agents including SGLT-2 inhibitors, GLP-1 receptor agonists, and DPP-4 inhibitors.
- Educate patients and caregivers to recognize nocturnal symptoms of hypoglycemia such as night sweats, nightmares, morning headaches, and confusion. Ensure caregivers are trained in the proper use of glucagon in case of severe hypoglycemia.
- For patients who have recurrent nocturnal hypoglycemia, ensure they eat an appropriate bedtime snack containing complex carbohydrates, and limit or avoid evening alcohol intake. Check bedtime and early-morning glucose (at 3 a.m. if needed) and use CGM with low-glucose alerts. CGM alone or in combination with an AID system has been shown to reduce hypoglycemia [50].

#### 2.3.3. Reactive or Postprandial Hypoglycemia

By definition, reactive hypoglycemia refers to hypoglycemia within 2 to 5 h after food intake. Suspected reactive hypoglycemia should be confirmed by the presence of Whipple’s triad before diagnostic work-up, which includes (1) a low plasma glucose level, (2) signs and symptoms in keeping with hypoglycemia, and (3) improvement in those signs and symptoms with increased plasma glucose levels.

Reactive hypoglycemia can arise from conditions that cause endogenous hyperinsulinism, including insulinomas, post-bariatric surgery hypoglycemia, and non-insulinoma pancreatogenous hypoglycemia. It may also occur secondary to critical illness, hepatic or renal dysfunction, hormonal deficiencies, non-diabetes medications, or non-islet cell tumors. The initial diagnostic approach should prioritize a comprehensive patient history, detailing the timing and characteristics of symptoms, current medications, underlying comorbidities, and any recent acute illnesses to inform and guide further evaluation.

Reactive hypoglycemia (RH) can be categorized into three different forms clinically:

1. Alimentary RH (at 120 min)
2. Idiopathic RH (at 180 min)
3. Late RH (240–300 min)

#### 2.3.4. Alimentary Reactive Hypoglycemia

Alimentary RH occurs primarily due to accelerated gastric emptying, leading to an exaggerated incretin response and increased levels of glucagon-like peptide-1 (GLP-1) and glucose-dependent insulinotropic peptide (GIP) that stimulate insulin secretion from pancreatic beta cells while simultaneously suppressing glucagon release. This ultimately results in early postprandial hypoglycemia. This form of reactive hypoglycemia can also occur in patients who have undergone vagotomy, gastrectomy, pyeloroplasty, or esophageal resection, as well as in those with peptic ulcer disease, renal glycosuria, or altered gastric motility.

#### 2.3.5. Idiopathic Reactive Hypoglycemia

Idiopathic RH is a diagnosis of exclusion and can otherwise be called functional reactive hypoglycemia. The pathophysiology remains unclear. One hypothesis is that it results from gastrointestinal dysfunction leading to relatively increased insulin secretion or enhanced insulin sensitivity. In some cases, patients may have adult-onset non-insulinoma pancreatogenous hypoglycemia, which is characterized by postprandial symptomatic hypoglycemia accompanied by hyperinsulinemia and elevated plasma C-peptide levels. Once the diagnosis is confirmed, other causes of reactive hypoglycemia should be ruled out.

#### 2.3.6. Late Reactive Hypoglycemia

Late RH occurs typically around 5 h postprandially. It is often triggered by delayed insulin response to a high-carbohydrate meal. Several factors can contribute to this condition, including insulin resistance or prediabetes. In these cases, delayed insulin secretion leads to late hyperinsulinemia in response to post-meal hyperglycemia, which ultimately results in hypoglycemia [51,52].

The oral glucose tolerance test (OGTT) remains the most commonly used method of diagnosing reactive hypoglycemia (RH). The mixed meal tolerance test (MMTT) can alternatively be performed to assess postprandial hypoglycemic symptoms. Unlike the OGTT, which involves ingestion of glucose alone, the MMTT includes protein, carbohydrates, and fat, providing a closer approximation of a typical meal in a patient’s daily diet. However, there is no standardized definition or protocol for the MMTT, and guidelines regarding meal composition and postprandial measurements are lacking. A diagnosis of RH also requires exclusion of other conditions that may influence hypoglycemic episodes, such as prior gastrointestinal surgery, peptic ulcer disease, hormonal disorders, and alcohol intake [53].

The cornerstone to managing reactive hypoglycemia is dietary modification. Patients are advised to consume smaller, more frequent meals (4–5 per day) that include a balanced mix of proteins, vegetables, and carbohydrates while avoiding carbohydrate-only snacks. Metformin may also provide benefit through several mechanisms: it reduces hepatic glucose production, inhibits gluconeogenesis and enhances glucose uptake in muscle, liver, and adipose tissue. These actions help moderate postprandial glucose excursions, thereby limiting hyperinsulinemia and lowering the risk of subsequent hypoglycemia [54,55].

Low-dose acarbose can be used effectively in reactive hypoglycemia that is refractory to conservative management.

In patients with late RH accompanied by impaired fasting glucose (IFG), metformin, thiazolidinediones (TZDs), DPP-4 inhibitors, and GLP-1 receptor agonists are other potential treatment options [56].

### 2.4. Autonomic Function

In T1D, both the loss of β-cell function and a defective α-cell response to falling glucose levels increase susceptibility to hypoglycemia. Older adults are particularly at risk due to age-related autonomic decline that limits their ability to generate an adequate counter-regulatory response. Additional factors such as greater glucose variability, previous hypoglycemic episodes, and impaired awareness of hypoglycemia further elevate the risk of severe events. This vulnerability is largely attributed to the development of hypoglycemia-associated autonomic failure (HAAF) [57].

The pathophysiology of HAAF includes defective glucose counter regulation (a failure to release glucagon due to impaired intra-islet insulin signaling and progressive reduction in epinephrine secretion) and hypoglycemia unawareness (due to diminished sympathoadrenal symptom generation and the resulting neurogenic symptom response). This creates a vicious cycle of hypoglycemia unawareness and recurrence, particularly in T1D and advanced T2D. While the exact mechanisms remain unclear, brain adaptations possibly involving altered metabolism or post-hypoglycemic glycogen supercompensation may underlie the shift in glycemic thresholds. This framework explains why hypoglycemia becomes more frequent as patients progress toward absolute insulin deficiency. Recognizing and reducing HAAF-related risk factors enables clinicians to individualize glycemic goals and lower the risk of hypoglycemia-related complications, including potential mortality [58].

Strategies such as CGM may help to reduce the risk of hypoglycemia but do not eliminate it entirely, as counterregulatory defects and hypoglycemia unawareness may persist. Additionally, interventions such as pancreatic islet transplantation have shown potential to restore counterregulatory and autonomic responses. However, evidence suggests that HAAF is partially reversible with meticulous hypoglycemia avoidance for 2–3 weeks. Therefore, clinicians should take these physiological limitations into account when selecting glucose-lowering therapies and setting individualized glycemic targets.

### 2.5. Cognitive and Mood Function

#### 2.5.1. Hypoglycemia and Cognitive Decline

Acute severe hypoglycemic episodes induce chronic subclinical brain damage, cognitive decline, and subsequent dementia. However, the impact of recurrent moderate hypoglycemia on long-term cognitive outcomes is not studied well. Proposed pathophysiological mechanisms include post-hypoglycemic neuronal injury, neuroinflammation, coagulation and endothelial dysfunction, and synaptic impairment of hippocampal neurons during hypoglycemic events [58]. In a population-based cohort study, individuals who experienced hypoglycemic episodes had a significantly higher risk of developing all-cause dementia, Alzheimer’s disease (AD), and vascular dementia (VaD) compared with those without such events (HR 1.254; 95% CI, 1.166–1.349; *p* < 0.001 for all-cause dementia; HR 1.264; 95% CI, 1.162–1.375; *p* < 0.001 for AD; HR 1.286; 95% CI, 1.110–1.490; *p* < 0.001 for VaD). Moreover, the association with dementia increased with the frequency of hypoglycemic episodes, with hazard ratios (HR) of 1.170 for one episode, 1.201 for two to three episodes, and 1.358 for more than three episodes [59].

Another population-based cohort study demonstrated a significant association between severe hypoglycemia and an increased risk of dementia, particularly Alzheimer’s disease, over a mean follow-up period of 6.9 ± 1.7 years. The risk of dementia rose with the number of severe hypoglycemic episodes, with hazard ratios of 1.54 (95% CI, 1.48–1.60) for one episode and 1.80 (95% CI, 1.66–1.94) for two or more episodes [60] resulting from recurrent hypoglycemic events in patients with diabetes.

A better understanding of the relationship between hypoglycemia and dementia can help to guide preventative strategies [61]. A recent study by Ye M. et al. demonstrated a clear dose–response relationship between the frequency of hypoglycemic episodes and the risk of cognitive impairment in individuals with type 2 diabetes [62]. Similarly, a 12-year prospective cohort study conducted by Yaffe et al. of 783 older adults with diabetes found that those who experienced hypoglycemia had twice the risk of developing dementia compared to those without such episodes [63]. Furthermore, national data indicate that nearly two-thirds of older adults in the U.S. with both diabetes and cognitive impairment are prescribed high-risk glucose-lowering agents, with about one-third using insulin [64].

To reduce the risk of hypoglycemia in older adults with diabetes and cognitive impairment, current guidelines recommend adopting more relaxed glycemic targets. In severe cases, this may involve foregoing strict glycemic control and avoiding medications with a high potential for hypoglycemia, such as sulfonylureas and insulin [65].

#### 2.5.2. Assessment of Cognitive Function

Cognition should be assessed at the time of diagnosis of hypoglycemia, especially in severe hypoglycemia, as well as in patients with recurrent hypoglycemia on an annual basis.

Effective assessment of mild cognitive impairment (MCI) in this population is critical for guiding management and preventing progression to dementia. In a pilot study conducted by the authors, the Montreal Cognitive Assessment (MoCA) appeared to be a more effective screening tool than the Standardized Mini-Mental State Examination (SMMSE) for detecting mild cognitive impairment (MCI) in individuals with diabetes [66]. However, the Quick Mild Cognitive Impairment (QMCI) screen has demonstrated comparable or even superior sensitivity and specificity relative to both the MoCA and MMSE [67]. In a separate study by Valles-Salgado M. et al., the diagnostic performance of several cognitive screening tools was compared, including the Mini-Mental State Examination (MMSE), Addenbrooke’s Cognitive Examination III (ACE-III) and its brief version (M-ACE), Memory Impairment Screen (MIS), Montreal Cognitive Assessment (MoCA), and Rowland Universal Dementia Assessment Scale (RUDAS). The results indicated that ACE-III and M-ACE had superior diagnostic accuracy for MCI compared with the other tests. MoCA and MMSE demonstrated adequate performance, while MIS and RUDAS showed limited diagnostic utility [68].

For predicting the progression from mild cognitive impairment (MCI) to dementia at baseline, MMSE scores show sensitivities ranging from 23% to 76% and specificities from 40% to 94%. For progression from MCI to Alzheimer’s dementia, baseline MMSE sensitivities range from 27% to 89%, and specificities from 32% to 90%. Given this variability, clinicians may prefer to use additional, more comprehensive assessments to guide patient management. Future research should evaluate whether tracking changes in MMSE scores over time provides a more reliable prediction of conversion from MCI to dementia than single baseline measurements [69], The DSM-V criteria (DSM V criteria (American Psychiatric Association. Diagnostic and statistical manual of mental disorders (DSM-V), American Psychiatric Association, Arlington (VA), 2013) are necessary for the diagnosis of dementia in addition to office- and bedside-based screening tests for cognitive impairment and the dementias like MMSE, MoCA and Mini-Cog [70].

Emerging evidence suggests that GLP-1 receptor agonists (GLP-1RAs) may have neuroprotective potential. Animal studies indicate that these agents can improve cognitive function, reduce cerebral amyloid-β accumulation in Alzheimer’s disease, modulate dopamine levels in Parkinson’s disease, and exert beneficial effects in brain ischemia. These findings suggest that GLP-1RAs could help preserve neuronal integrity and potentially improve outcomes in diabetes-related cognitive decline [71].

#### 2.5.3. Hypoglycemia and Mood

Hypoglycemia is associated with negative effects on mood and energy levels [72]. The Diabetes Social Support Questionnaire (DSSQ) and the Problem Areas in Diabetes (PAID) scale, a 20-item questionnaire, can be used to assess emotional distress related to diabetes management, including feelings of guilt, frustration, and anxiety [73,74]. Compared to other tools, the PAID scale captures a broader range of emotional challenges, particularly those related to diet and diabetes complications, and shows a stronger correlation with depressive symptoms and maladaptive coping strategies [75]. Caregivers and family members of individuals experiencing hypoglycemia often face the resulting mood disturbances, which can strain relationships and further affect overall quality of life [76]. The 2014 InHypo-DM study in Canada studied the emotions experienced by patients regarding hypoglycemia. The predominant emotion experienced by patients was “fear”. Others include loss of control over one’s life, anxiety, frustration and death in bed syndrome. However, after learning about hypoglycemia management and preventive strategies, their frustration waned and was replaced by feelings of confidence, self-efficacy, and hope.

As suggested by recent recommendations, physicians should screen for fear of hypoglycemia during the early months after diagnosis of diabetes and promote self-confidence in the management of hypoglycemia, called “hypoglycemia confidence”. Hypoglycemia confidence can be achieved by offering specific learning opportunities that foster personal awareness about hypoglycemia, its symptoms, and management. Other adaptive methods include promoting a positive attitude and sense of resilience while having diabetes and receiving physiological support through caring family and friends [77,78].

### 2.6. Renal, Liver and Pancreatic Function

#### 2.6.1. Renal Function and Hypoglycemia

In patients with diabetes and advanced chronic kidney disease (CKD), glucose and insulin metabolism are significantly disrupted, leading to an increased risk of hypoglycemia. This is due to multiple factors, including impaired renal insulin clearance, defective insulin degradation due to uremia, increased erythrocyte glucose uptake during hemodialysis, impaired counter-regulatory hormone responses (cortisol, growth hormone, epinephrine), nutritional deprivation, and variable exposure to oral anti-hyperglycemic agents that potentiate the effects of exogenous insulin [79]. Hemodialysis further complicates glucose regulation. Alternative glycemic biomarkers such as glycated albumin or fructosamine are even less reliable than HbA1c. Measuring blood glucose control with HbA1c is problematic in CKD due to reduced erythropoiesis, though it remains the standard biomarker for lack of more reliable alternatives [80].

Diabetes increases the risk of chronic kidney disease (CKD) by approximately 2.6-fold through the development of diabetic nephropathy [81]. This is exacerbated by suboptimal glycemic control, particularly in patients who have had diabetes for a long period of time. Assessing renal function in the elderly presents unique challenges, as regular blood chemistry markers like creatinine and urea often do not provide accurate information in isolation. Instead, more specific renal function parameters such as creatinine clearance, inulin clearance, or cystatin C levels are preferred. Creatinine and creatinine-based markers have limitations in older adults due to sarcopenia, which reduces muscle mass and affects serum creatinine levels.

Several formulas are used to estimate creatinine clearance. The Modification of Diet in Renal Disease (MDRD) formula is commonly used in clinical laboratories, while the Cockcroft–Gault formula is frequently employed in clinical trials. The MDRD Study equation (requires only serum Cr and patient age) is the most commonly used GFR formula, automatically calculated by labs. The modified Cockcroft–Gault formula gives more accurate eGFR estimates, as it adjusts for BMI and takes body surface area into account [82]. The CKD-EPI (Chronic Kidney Disease Epidemiology Collaboration) formula is often considered more accurate at higher glomerular filtration rates (GFR) and is particularly useful for identifying early-stage CKD. While inulin clearance remains the gold standard for calculating GFR, also cystatin C-based methods provide a more precise assessment of kidney function.

#### 2.6.2. Liver Function and Hypoglycemia

The liver is essential for maintaining glucose homeostasis through glycogen storage, gluconeogenesis, and glucose metabolism. It is important to determine the extent of liver damage and address the underlying cause, such as alcohol use or viral hepatitis that could lead to hypoglycemia in older adults with DM [83,84]. This includes the assessment of clinical signs of liver disease, including jaundice, ascites, and encephalopathy, as well as liver function tests and imaging such as ultrasound, CT, or MRI. Insulin resistance is common in patients with cirrhosis. In addition, reduced insulin clearance due to loss of hepatic mass and portosystemic shunting places these patients at increased risk of hypoglycemia. Cirrhotic patients also have reduced glycogen storage and impaired gluconeogenesis, limiting their ability to counter hypoglycemia. The metabolism and excretion of oral hypoglycemic drugs may be significantly affected by hepatic dysfunction. Patients with cirrhosis exhibit reduced hepatic metabolism, altered intestinal mucosal permeability, and changes in intestinal microbiota. Cytochrome P450 activity is decreased, and hypoalbuminemia with fluid retention increases the free plasma concentration of protein-bound drugs. Cholestasis can further impair drug clearance. Collectively, these factors influence drug absorption, distribution, bioavailability, metabolism, and elimination. Therefore, glycemic targets and therapeutic regimens should be individualized to minimize hypoglycemic events in patients with liver dysfunction.

Diagnosis and monitoring of DM in patients with cirrhosis are challenging, as fasting blood sugar values may be low, and HbA1c may be falsely low due to hemolysis secondary to hypersplenism or gastrointestinal blood loss. Given these limitations, an oral glucose tolerance test (OGTT) should be considered for diagnosis. The management of diabetes in decompensated cirrhosis is further complicated by hepatic encephalopathy, altered drug metabolism, frequent renal dysfunction, risk of lactic acidosis, and associated malnutrition and sarcopenia. Frequent self-monitoring of blood glucose may be required and should ideally be performed preprandially and 2 h postprandially. CGM can provide comprehensive information on glucose fluctuations and may be considered in patients on insulin or those with recurrent hypoglycemia.

The following lifestyle strategies and therapeutic regimens can be used to minimize the risk of hypoglycemia in patients with cirrhosis:

- Dietary modification and physical activity are the cornerstones of diabetes management in patients with cirrhosis. Nutritional recommendations should aim to achieve glycemic control while preventing or worsening sarcopenia and malnutrition. Multiple small, frequent meals are recommended to avoid prolonged fasting periods.
- Many anti-hyperglycemic agents are either contraindicated or should be used with caution in patients with cirrhosis. There have been concerns regarding the safety of metformin, leading many clinicians to avoid its use due to fear of metformin-associated lactic acidosis (MALA). However, MALA is extremely rare, with an estimated incidence of <10 per 100,000 patient-years in those without significant renal impairment. The risk is higher in patients who develop acute renal dysfunction due to dehydration, vomiting, or diarrhea, particularly in elderly patients with low glomerular filtration rates. However, even in patients with renal impairment and estimated GFRs of 10–30 mL/min/1.73 m^2^, lower doses (500–1500 mg/day) may be safe.
- Several other anti-hyperglycemic drug classes, such as meglinitides, are known to cause hypoglycemia due to reduced hepatic clearance and should be avoided. Thiazolidinediones, particularly troglitazone, are hepatotoxic. Although other thiazolidinediones are less hepatotoxic, there are case reports of liver injury with rosiglitazone and pioglitazone. Additionally, these agents cause fluid retention and should therefore be avoided in cirrhosis.
- Pharmacokinetic studies of DPP-4 inhibitors following single-dose administration in patients with chronic liver disease (CLD) have shown no clinically significant effects requiring dose adjustment. However, due to a higher risk of hepatotoxicity, vildagliptin should be avoided, while other DPP-4 inhibitors may be used cautiously. GLP-1 receptor agonists should be used with caution in CLD because of limited safety and efficacy data in advanced liver disease. All SGLT-2 inhibitors are metabolized in the liver. They may be used in Child–Pugh A cirrhosis, used with caution in Child–Pugh B, and should be avoided in Child–Pugh C cirrhosis.
- Predicting insulin requirements in patients with CLD can be challenging. In decompensated cirrhosis, insulin requirements may be reduced due to decreased hepatic insulin breakdown and impaired gluconeogenesis, while insulin resistance may necessitate higher doses in other patients. Basal insulin should be initiated at 0.1–0.2 units/kg/day as a single daily dose of a long-acting insulin analogue, with a 25% dose reduction. If glycemic targets are not met, premeal rapid-acting insulin may be added, starting at 4 units or 10% of the basal dose with the meal associated with the greatest postprandial glucose rise. The dose may be titrated by 1–2 units every 3–4 days based on blood glucose readings.

Although insulin is often recommended as the preferred therapy in decompensated cirrhosis, it is associated with weight gain, hypoglycemia and an increased risk of hepatocellular carcinoma. Therefore, insulin therapy should be reserved for patients who are unable to tolerate antihyperglycemic agents or who fail to achieve adequate glycemic control [85,86].

#### 2.6.3. Pancreatic Function and Hypoglycemia

Type 3c DM is referred to as pancreatogenic DM. Hypoglycemia in type 3c DM results from the combined failure of several protective mechanisms:

- Pancreatic inflammation, such as in chronic or severe acute pancreatitis, leads to destruction of islet α-cells, causing loss of glucagon—the principal counter-regulatory hormone required to stimulate hepatic glycogenolysis and gluconeogenesis during falling glucose levels.
- Loss of pancreatic polypeptide from islet alpha cells further disrupts hepatic insulin regulation, resulting in inappropriate suppression of hepatic glucose output. At the same time, patients often remain insulin-sensitive, so even relatively small doses of exogenous insulin can cause hypoglycemia.
- Maldigestion and malabsorption cause unpredictable carbohydrate absorption and impaired incretin responses, leading to mismatches between insulin administration and nutrient availability.

In patients with chronic pancreatitis, the initial evaluation should include fasting glucose and HbA1c levels. If either is impaired, further evaluation with a standard 75 g oral glucose tolerance test (OGTT) is recommended, along with pancreatic imaging and differentiation from type 1 DM by excluding the presence of islet autoantibodies. The measurement of a C-peptide level along with plasma glucose can also estimate the patient’s endogenous insulin production. An inappropriately normal or a low C-peptide in the setting of hyperglycemia may indicate a relative or absolute insulin deficiency, respectively, as a result of the pancreatitis.

Type 3c DM can be established by measuring the pancreatic polypeptide (PP) response to mixed-nutrient ingestion. Mixed-nutrient ingestion can be standardized to 12 ounces of Boost High Protein^®^ or an equivalent formulation and administered with prescribed pancreatic enzymes. Non-diabetic subjects demonstrate a 4–6-fold increase over basal PP levels, whereas patients with chronic pancreatitis-associated diabetes demonstrate less than a doubling of their basal values.

In chronic pancreatitis-associated diabetes, when hyperglycemia is mild (HbA1c < 8%), oral hypoglycemic therapy with metformin is recommended. Sulfonylureas and meglitinides should be avoided. Incretin-based therapies, including GLP-1 analogues and DPP-4 inhibitors, have been associated with cases of drug-induced pancreatitis. Until more data are available, their use remains controversial.

Insulin therapy is preferred in most patients with advanced type 3c DM. Multidose basal–bolus insulin regimens should follow guidelines for the treatment of type 1 DM. Insulin-induced hypoglycemia and insulin regimens with a lower risk of hypoglycemia are discussed under “Medication Function” in this article.

The introduction of new hepatoprotective diabetes drugs into clinical practice, including tirzepatide, SGLT2i, and GLP-1 RA, sets the stage for future trials to investigate the ideal therapeutic regimen for people with T2D and compensated cirrhosis. Metformin, sodium-glucose co-transporter-2 inhibitors (SGLT2is), and glucagon-like peptide-1 receptor agonists (GLP-1 RAs) are promising treatment options for patients with T2D and compensated liver cirrhosis, offering good glycemic control with minimal risk of hypoglycemia, while their pleiotropic actions confer benefits on non-alcoholic fatty liver disease (NAFLD) and body weight, and decrease cardiorenal risk [87,88].

### 2.7. Social Function

Hypoglycemia can significantly impact social function. The fear of hypoglycemia, particularly among insulin users, may lead to avoidance of social situations and reduced participation in activities. Additionally, low socioeconomic status, difficulties in obtaining food and groceries, and challenges in cooking due to cognitive or physical disabilities can further increase the risk of hypoglycemia [89,90,91,92,93,94].

The social impact of hypoglycemia has not been extensively studied, and it is sometimes regarded as an unavoidable consequence of tight glycemic control aimed at reducing microvascular complications. However, recurrent hypoglycemic episodes can interfere with optimal blood sugar management and negatively affect quality of life, contributing to depressive symptoms, heightened anxiety and impairments in activities such as driving and work [95,96,97,98,99,100]. Diabetes-related distress increases with the frequency of hypoglycemic episodes.

A comprehensive assessment should include evaluation of social functioning (Table 3), particularly the impact of nocturnal and severe hypoglycemia on health-related quality of life [93,94,95,96,97,98,99]. The Problem Areas in Diabetes (PAID) scale is a 20-item questionnaire designed to measure emotional distress associated with diabetes management, including feelings such as guilt, frustration, and anxiety. The Diabetes Distress Scale (DDS), a 17-item questionnaire, evaluates emotional distress associated with living with diabetes, including concerns about the future, self-blame, and feelings of isolation [100,101].

Diabetes distress can be alleviated through structured diabetes education and self-management support. Such interventions often include training in self-care behaviors, including healthy eating, regular physical activity, blood glucose monitoring and medication management. Physical activity has been shown to improve both physical and psychological health, including reducing diabetes-related distress. Peer support can further mitigate feelings of isolation and promote overall wellbeing [102]. Cognitive behavioral therapy (CBT) has also been shown to reduce diabetes-related distress and improve quality of life. Likewise, mindfulness-based interventions focused on present-moment awareness and non-judgmental acceptance can reduce stress and enhance psychological wellbeing [103]. Education programs can further support patients using smartphone applications and telemonitoring technologies [104].

### Social Determinants of Health-Related Factors in Relation to Hypoglycemia

Further research is needed to assess the effect of age-related social needs on severe hypoglycemia in older populations with diabetes. Results from a US-based survey (mean respondent age: 58 years) indicate inverse relationships between educational attainment, health literacy, and annual income, with risks for severe hypoglycemia [105,106]. Food insecurity has also been shown to double hypoglycemic event rates [107]. Other social factors that may predict the occurrence of severe hypoglycemia among older adults include area-level deprivation, inequitable care provision, social isolation, and certain cultural practices such as Ramadan.

### 2.8. Physical Function

Hypoglycemia causes dizziness, temporary loss of coordination, accelerated muscle loss (sarcopenia), reduced muscle strength and falls. It can affect activities of daily living (ADL), and it can be assessed by Katz ADL [108]. It can affect mobility and balance, which can be assessed by the Timed -Up and Go test (TUG) [109], 4 Meter Walk Test, Tinetti Gait and Balance and Berg Balance assessments [110,111,112]. It is crucial to assess patients for risk of falls and loss of independence in their day-to-day activities.

Hypoglycemia impairs neuromuscular performance, postural control, endurance and functional independence, increasing the risk of falls, injuries and activity restriction. A meta-analysis to identify risk factors for falls in older adults with T2D showed an increased risk of falls in diabetic vs. non-diabetic adults secondary to presence of neuropathy, impaired vision and decreased physical performance. Therefore, physical function in adults with hypoglycemia requires thorough assessment of baseline capacity. The parameters and assessment tools to determine baseline physical function are discussed in Table 4 [113,114].

#### 2.8.1. Strategies to Overcome Physical Function Decline Due to Recurrent Hypoglycemia

Hypoglycemia in adults causes an accelerated decline in muscle strength and functional status. The benefits of resistance training for all adults include improvements in muscle mass, strength, physical function, mental health, bone mineral density, insulin sensitivity and cardiovascular health.

However, exercise routines or strenuous activities in adults with a history of hypoglycemia should be planned and monitored closely. Strategies to avoid hypoglycemia include checking blood glucose levels before and after exercise, consuming complex carbohydrates with protein if blood glucose is less than 100 mg/dL (5.6 mmol/L) before physical activity, dose-reducing rapid-acting insulin prior to exercise, and scheduling insulin administration to avoid peak insulin action during exercise.

Glycemic targets should be individualized to avoid overly strict glycemic control. This includes adjusting insulin regimens, de-intensifying hypoglycemia-causing medications (e.g., insulin, sulfonylureas, or meglitinides), switching to medication classes with a low risk of hypoglycemia, and using continuous glucose monitoring (CGM) with alert systems. These strategies, in turn, minimize recurrent hypoglycemia, which would ultimately minimize its impact on neuromuscular and cognitive functions that can limit physical performance.

Safety strategies should be implemented to reduce the risk of falls due to hypoglycemia, including installing grab bars, using appropriate supportive footwear and carrying fast-acting glucose at all times.

Adequate and appropriately timed nutrition also supports glucose stability and physical capacity. This includes balanced meals containing carbohydrates, fats, and proteins, bedtime snacks to prevent nocturnal hypoglycemia, and sufficient protein intake to prevent sarcopenia.

In conclusion, these patients require a multidisciplinary team approach involving physicians to optimize medications and assess the frequency and severity of hypoglycemic episodes. Occupational and physical therapists may also be consulted for functional/physical training and fall prevention, and dietitians for individualized meal planning [115,116].

#### 2.8.2. Hypoglycemia and Frailty

Frailty is a common geriatric syndrome that can amplify diabetes-related complications in older people. Frailty has been described as “a condition characterized by loss of biological reserves across multiple organ systems and vulnerability to physiological decompensation after a stressor event” [117,118]. Weight loss and malnutrition among frail older adults with diabetes significantly heighten the risk of hypoglycemia. The ADVANCE trial demonstrated that frailty was associated with a higher risk of vascular events, all-cause mortality, cardiovascular mortality, and hypoglycemia [119]. Hypoglycemia increases the risk of incident frailty by 44% (HR 1.443, 95% CI 1.01–2.05) [120]. Glycemic control in older adults should therefore be individualized according to the degree of frailty. In those with moderate or advanced frailty and reduced life expectancy, strict glycemic control is not recommended, as it may increase the risk of hypoglycemia [121,122].

#### 2.8.3. Frailty Phenotypes

Frailty in older adults has a wide metabolic spectrum ranging from a sarcopenic–obese phenotype to an anorexic–malnourished phenotype. In individuals with the sarcopenic–obese phenotype, diabetes typically progresses more rapidly, necessitating intensification of glucose-lowering therapy along with careful management of cardiovascular risk factors. Conversely, the anorexic–malnourished phenotype is characterized by significant weight loss and reduced insulin resistance, leading to a slower diabetes trajectory.

For individuals with the sarcopenic–obese phenotype, early initiation of SGLT-2 inhibitors or GLP-1 receptor agonists is particularly reasonable due to their weight-reducing effects and cardio-renal protective properties. Meanwhile, in the anorexic–malnourished phenotype, long-acting insulin analogues may be preferred early on because of their anabolic effects, potential to promote weight gain, simple dosing regimen, and convenience of once-daily administration [123].

#### 2.8.4. Antihyperglycemic Pharmacotherapy and Frailty

In frail older adults with T2D, metformin remains the preferred first-line therapy due to its favorable safety profile and low risk of hypoglycemia. A cross-sectional study by Baskaran et al. showed metformin exposure is associated with low risk of frailty. Medications with a higher hypoglycemia risk, such as sulfonylureas and meglitinides, should generally be avoided in this population. In contrast, SGLT-2 inhibitors and GLP-1 receptor agonists can be safely used in frail adults, offering additional cardiovascular and renal benefits along with minimal hypoglycemia risk [124,125].

## 3. Management of Hypoglycemia in Older Adults

### 3.1. Immediate Management

#### 3.1.1. Conscious Patient

To treat low blood sugar, the American Diabetes Association advises using the 15-15 Rule: consume 15 g of fast-acting carbs, wait 15 min to recheck your levels, and repeat if your reading remains under 70 mg/dL.

#### 3.1.2. Unconscious Patient

In an unconscious person with severe hypoglycemia, if there is no intravenous access, 1 mg of glucagon should be administered via subcutaneous or intramuscular injection, or 3 mg administered via intranasal spray. Glucagon mobilizes glycogen from the liver and stimulates glycogenolysis, generating glucose in rapid fashion. In situations such as alcohol ingestion or prolonged fasting, when liver glycogen stores are depleted, caregivers or support persons should immediately contact emergency services and inform the diabetes healthcare team as soon as possible. If intravenous access is available, 10–25 g of glucose of D50W should be administered over 1–3 min. Care must be taken to avoid extravasation of 50% dextrose, as this can lead to tissue necrosis. After hypoglycemia is corrected, the individual should consume their usual meal or snack to prevent recurrence. If the next meal is more than an hour away, a snack containing 15 g of carbohydrate along with a protein source is recommended. Additionally, it is crucial for individuals at risk of severe hypoglycemia to have support persons trained in glucagon administration [126].

### 3.2. Subsequent Management of Hypoglycemia

Use antihyperglycemic medications with less hypoglycemic risk, such as metformin, DPP-4 inhibitors, thiazolidines (TZD), GLP-1 receptor agonists (GLP-1 RAs), and SGLT-2 inhibitors [127].

Hypoglycemia risk with DPP-4 inhibitors: DPP-4 inhibitors carry a low risk of hypoglycemia, although saxagliptin has been associated with a small, non-significant increase in heart failure risk [128]. TZDs may increase the risk of hypoglycemia and can cause fluid retention, so they should be used with caution in diabetic patients with heart failure [129].

#### 3.2.1. Hypoglycemia Risk with SGLT-2 Inhibitors

The overall risk of hypoglycemia resulting from SGLT-2 inhibitor use is low, and several meta-analyses revealed that the risk of hypoglycemia due to SGLT-2 inhibitor monotherapy was similar to that of placebos. On the other hand, a cohort study from Japan showed the risk may be higher when combining SGLT-2 inhibitors with sulfonylureas (HR1.44, 95% CI 1.02–2.03)/ and/or insulin (HR 1.44, 95% CI 1.02–2.03), but the risk was low in concomitant metformin users (HR 0.52; 95% CI 0.37–0.74) [130]. SGLT-2 inhibitors can cause an increased risk of urinary tract infections, euglycemic diabetic ketoacidosis, dehydration and falls so should be used with caution in older adults [131].

#### 3.2.2. Hypoglycemia Risk with GLP-1

Glucagon-like peptide-1 (GLP-1), a gut-derived peptide secreted by intestinal cells in response to glucose ingestion, lowers blood glucose by enhancing insulin secretion via activation of the GLP-1 receptor. Based on this mechanism, a class of drugs called glucagon-like peptide-1 receptor agonists (GLP-1RAs) was developed to improve glycemic control in patients with type 2 diabetes. GLP-1RAs are most often delivered as long-acting agents—dulaglutide, semaglutide and liraglutide, as short-acting agents, have fallen out of favor. Beyond their potent glucose-lowering effects, GLP-1RAs have demonstrated cardiovascular benefits in major outcome trials, including LEADER, SUSTAIN-6, Harmony Outcomes, and REWIND. Although they have a low risk of hypoglycemia in isolation, clinicians should exercise caution when prescribing GLP-1RAs to patients at high risk for hypoglycemia if given with insulin or sulfonylureas [132].

Tirzepatide is a next-generation incretin therapy that activates both GIP and GLP-1 receptors. Due to its glucose-dependent mechanism, it carries a low risk of hypoglycemia when used as monotherapy or in combination with metformin. It primarily stimulates insulin secretion and suppresses glucagon only when blood glucose is elevated. However, the risk of hypoglycemia rises substantially when tirzepatide is combined with insulin or insulin secretagogues such as sulfonylureas. In the SURPASS-5 trial, hypoglycemia occurred in less than 1% of patients on tirzepatide monotherapy but increased to nearly 19% when combined with basal insulin. These results underscore the need for careful dose adjustment of concurrent glucose-lowering therapies and vigilant blood glucose monitoring when tirzepatide is added to combination regimens [133]. For patients with weight loss, gastroparesis and anorexia, GLP1 receptor agonists should be avoided.

#### 3.2.3. Hypoglycemia Risk with Insulin

In general, a long-acting basal insulin is associated with a lower risk of hypoglycemia compared to using bolus (mealtime) insulin or premixed insulin. Second-generation basal insulin analogues (e.g., insulin degludec, insulin glargine U-300) carry a lower hypoglycemia risk compared to earlier basal insulins (like NPH insulin, Insulin detemir and Insulin glargine U-100) [134,135].

#### 3.2.4. Severe Hypoglycemia Treatment—Dasiglucagon

A recent glucagon receptor agonist, dasiglucagon, when used in the management of severe hypoglycemia, has been hypothesized to have a role in mitigating AD neuropathology, although this has not been evaluated in preclinical or clinical studies [136].

### 3.3. Non-Pharmacologic Management

#### 3.3.1. Role of Healthcare Providers

Assessment of hypoglycemia should be part of every patient visit and is a standard of diabetes care. The role of healthcare providers include the following steps shown in Table 5 [11].

Both American Diabetes Care and Diabetes Canada recommend choosing medications that demonstrate both cardiac and renal protection and carry a low risk for hypoglycemia. Patient care should be individualized.

#### 3.3.2. Patient Education and Empowerment

All patients and their caregivers should receive education regarding glucose monitoring (POC capillary glucose monitoring or CGM), dietary intake, alcohol, and exercise, as well as detailed information about antihyperglycemic medications, insulin dosing and peak effects, and driving. One of the main reasons for recurrent hypoglycemia is lack of understanding of diabetes management and therapeutic regimens. Subjective recognition of hypoglycemia symptoms is fundamental for effective self-management.

- In elderly patients, there is concern for hypoglycemia when blood glucose reaches 70 mg/dL (4 mmol/L).
- At this threshold, or at the onset of early hypoglycemia symptoms, patients should promptly take rapid-acting glucose.
- The “rule of 15” is a practical guide for self-treatment: consuming 15 g of rapid-acting glucose typically raises blood glucose by approximately 50 mg/dL (2.8 mmol/L) within 15 min.
- Patients should always carry a source of rapid-acting glucose with them.
- All patients should wear a medical alert bracelet.
- Those with hypoglycemia unawareness should also have access to a glucagon kit or glucagon nasal spray, and family members or caregivers should be trained in its proper administration.
- Blood glucose should be checked before driving, with a recommended minimum level of 90 mg/dL (5 mmol/L). Patients should keep snacks and glucose tablets available in the car.
- Physicians should follow local regulations regarding reporting individuals deemed unfit to drive [137].

## 4. Management of Different Types of Hypoglycemia

### 4.1. Fasting Hypoglycemia

Fasting hypoglycemia occurs after prolonged periods without food intake, typically more than 8 h. It is seen with insulinomas and other pathologies such as adrenal insufficiency, hepatic dysfunction, or inborn errors of metabolism. Acute episodes are treated with rapid administration of glucose, either orally or intravenously, and in severe cases, glucagon may be used to restore blood sugar levels. Acute management has been discussed in detail under the heading “General Management of Hypoglycemia in Older Adults”. Once stabilized, patients should undergo biochemical evaluation to determine the etiology. If an insulinoma or other tumor-induced cause of endogenous hyperinsulinemia is found, surgical resection is often curative. In other causes like adrenal insufficiency or liver failure, hormone replacement or management of liver function is essential. Long-term strategies include frequent, balanced meals rich in complex carbohydrates, avoiding prolonged fasting, and regular monitoring to prevent recurrence.

### 4.2. Postprandial Hypoglycemia

Postprandial hypoglycemia, often called reactive hypoglycemia, is a drop in blood sugar that occurs within 2 to 5 h after eating. It is typically caused by an overproduction of insulin in response to a meal (often those high in simple carbohydrates), and can cause symptoms like shakiness, confusion, and a racing heart.

### 4.3. Nocturnal Hypoglycemia

Nocturnal hypoglycemia can be prevented by (a) using bed-time snacks, (b) avoiding intermediate-acting insulin at supper or bedtime, and (c) using long-acting insulin analogues such as detemir or glargine, as these are safer than intermediate insulin NPH and have minimal peaks during their duration of action. Daytime hypoglycemia can be reduced by using rapid-acting insulin analogues such as lispro, aspart, or glulisine as preprandial insulin instead of regular insulin. The use of premixed insulin formulations or prefilled insulin pens may also help minimize dosing errors, although they offer less dosing flexibility [138].

### 4.4. Refractory Hypoglycemia

Refractory hypoglycemia’s can be treated with Octreotide therapy [139,140].

## 5. Other Considerations in the Management of Hypoglycemia

### 5.1. Fear of Hypoglycemia

Fear of hypoglycemia (FOH) is a common phenomenon among individuals with diabetes who experience or are at risk of hypoglycemia. It is characterized by feelings of tension, anxiety, and discomfort, often accompanied by physical symptoms such as palpitations, shortness of breath, and hand tremors. Many patients who have experienced hypoglycemia develop this fear, which can lead them to intentionally maintain higher blood glucose levels to avoid future episodes. However, this compensatory behavior may result in poorer glycemic control and increase the risk of long-term diabetes complications [141]. Recurrent hypoglycemic episodes can reinforce and heighten FOH. Individuals affected by this fear often lose confidence in their body’s ability to regulate glucose and may doubt their capacity to respond appropriately in situations such as sleeping, driving, exercising, or engaging in social activities [142].

#### 5.1.1. Methods of Measuring Levels of FOH

The first tool developed to assess levels of fear of hypoglycemia (FOH) was the Hypoglycemia Fear Survey (HFS), created by Cox et al. and psychometrically validated in 1987 [143]. Other instruments used to measure FOH include the Quick Screening for Fear of Hypoglycemia (QSFH) and the 15-item Fear of Hypoglycemia Scale (FH-15) [144,145].

#### 5.1.2. Therapies for FOH

Psychoeducational interventions focused on hypoglycemia, such as Hypoglycemia Anticipation, Awareness and Treatment Training (HAATT), Blood Glucose Awareness Training II (BGAT-2), and the Hypoglycemia Prevention and Optimization Study (HyPOS), have been developed globally. These interventions aim to enhance patients’ daily self-observation and self-management skills, for example, by maintaining a hypoglycemia diary. They also incorporate group sessions that educate participants on proper nutrition, physical activity, insulin therapy, and the management of acute diabetes complications, particularly diabetic ketoacidosis [146,147,148,149,150].

The introduction of new glucose monitoring technologies such as CGM has enabled more precise glucose tracking and improved metabolic control in individuals with diabetes. Their use in diabetes management may also help to alleviate FOH [151].

### 5.2. Hypoglycemia Distress

Hypoglycemia can impair cognitive function, so it is important to assess cognitive abilities such as spatial ability that can be affected by low blood sugar. Hypoglycemia can affect mood and emotional state, leading to irritability, anxiety, or depression. Assessing these factors is important for understanding the overall impact of hypoglycemia on social functioning [152,153].

Hypoglycemia distress is an important psychosocial barrier in management. A key priority is providing guidance when individuals report high levels of both distress and depressive symptoms. The first part of addressing hypoglycemia distress occurs with a detailed discussion with the patient, family, and caregiver. Providing emotional support is very important during this process. Diabetes education and support for postgraduate trainees may help the future generation of healthcare professionals adequately address these issues [154]. The primary psychological approaches explored for managing these mental health conditions include cognitive behavioral therapy, cognitive conceptualization, dialectical behavior therapy, relational therapy, and psychoeducational interventions [155].

### 5.3. Impaired Awareness of Hypoglycemia

Impaired awareness of hypoglycemia (IAH) is a condition characterized by a reduced or absent ability to perceive the typical symptoms of low blood glucose [156]. A recent study reported the overall prevalence of IAH to be 17.6% (95% CI: 14.9–20.3%), with rates being higher among individuals with type 1 diabetes compared to those with T2D and the lowest prevalence observed in Europe [157]. Among people with diabetes undergoing hemodialysis, the prevalence of IAH was 23.2%. Hypoglycemia awareness can be challenging, as some individuals may be alerted to low glucose readings by continuous glucose monitoring systems before experiencing any physical symptoms [158].

IAH was particularly common in individuals who reported fear of hypoglycemia or had a history of severe episodes. These findings underscore the unmet clinical needs and disparities in access to diabetes technology among insulin-treated patients with diabetes on hemodialysis. With consistent avoidance of hypoglycemic episodes over time, awareness of low blood glucose can often be partially or fully restored [159].

Current strategies to improve hypoglycemia awareness include structured patient education, psychoeducational interventions, the use of advanced glycemic control technologies, pancreas or islet transplantation, and pharmacological therapy [160]. A recent review evaluated the efficacy and feasibility of various pharmacologic interventions for preventing and managing IAH in both clinical and preclinical studies. The agents discussed included N-acetylcysteine, GABA-A receptor blockers, opioid receptor antagonists, AMP-activated protein kinase (AMPK) agonists, potassium channel openers, dehydroepiandrosterone, metoclopramide, antiadrenergic agents, antidiabetic medications, and glucagon [161]. The management of IAH begins with its identification during clinical assessment, which should include a thorough review of the frequency of hypoglycemic episodes and an evaluation of the individual’s ability to recognize them [162].

## 6. Technology in the Management of Hypoglycemia

New technologies, such as continuous glucose monitors (CGMs) and automated insulin delivery (AID) systems, referred to as the artificial pancreas, have significantly improved the management of hypoglycemia.

CGMs provide continuous tracking of glucose levels and alert individuals when levels begin to drop, whereas AID systems automatically adjust insulin delivery to maintain glucose within a safe range. Over recent decades, the integration of these technologies has transformed diabetes management by enabling tighter glucose regulation and minimizing hypoglycemia risk [163]. Future studies are needed to explore the psychosocial effects of these technologies in greater depth [164]. Additionally, advanced therapeutic tools such as insulin pumps, bolus calculators, and low-glucose suspend features, especially when used in combination, may further help reduce the fear of hypoglycemia (FOH) [165]. A recent randomized controlled trial showed that an intervention based on the behavior change wheel (BCW) to reduce fear of hypoglycemia (FoH) behavior in individuals with T2D was effective [166].

## 7. Glycemic Targets for Patients with Hypoglycemia

Less stringent glycemic targets may be considered in older adults with diabetes who are on antihyperglycemic agents that carry a risk of hypoglycemia, particularly in those with dementia, frailty, or impaired hypoglycemia awareness.

1. Functionally dependent: Target HbA1c of 7.1–8.0%
2. Frail and/or with dementia: Target HbA1c of 7.1–8.5%
3. End of life: Routine HbA1c measurement is not recommended. Focus on avoiding symptomatic hyperglycemia and preventing hypoglycemia.

For instance, while most guidelines recommend a target HbA1c of <7% for the general adult population, targets are more relaxed for frail older adults. The American Geriatrics Society suggests an A1c < 8%, the Veterans Affairs and Department of Defense recommend 8–9%, and the American Diabetes Association advises “less stringent glycemic goals” without specifying exact targets. Despite some ambiguity in the current guidelines, the general consensus supports a loose management strategy for these populations [167].

The Canadian Diabetes Association (now Diabetes Canada) advises setting individualized glycemic targets for frail older adults, with a focus on preventing hypoglycemia rather than strictly achieving standard targets. Frailty—characterized by unintentional weight loss, fatigue, weakness, and low physical activity—warrants aiming for an A1C range of 7.1% to 8.5% [168].

Glycemic targets in older adults should be individualized, mainly focusing on minimizing hypoglycemia [169]. According to recent ADA recommendations, older adults with diabetes who are otherwise healthy, have few stable comorbidities, and retain intact cognitive and functional abilities should aim for lower glycemic targets, such as an HbA1c of <7.0–7.5% (<53–58 mmol/mol) and/or a time in range (TIR) of 70–180 mg/dL (3.9–10.0 mmol/L) of approximately 70%, with time below range (<70 mg/dL [3.9 mmol/L]) limited to ≤4% when using CGM.

For older adults with multiple medical comorbidities, who are clinically heterogeneous with varying life expectancy, glycemic goals should be individualized with an emphasis on hypoglycemia prevention. Less stringent targets, such as a HbA1c < 8.0% (<64 mmol/mol) and/or TIR of ∼50% within 70–180 mg/dL (3.9–10.0 mmol/L), with time below range < 70 mg/dL (<3.9 mmol/L) of <1%, are recommended for those with significant cognitive or functional impairment, frailty, severe comorbidities, or an unfavorable risk–benefit profile for diabetes medications.

For older adults with very complex health needs or poor functional baselines, intensive glycemic control provides minimal benefit. In these cases, clinicians should prioritize preventing hypoglycemia and symptomatic hyperglycemia rather than pursuing stringent glycemic targets [170].

Overall, these recommendations emphasize the need for individualized targets, with less stringent goals often appropriate for older adults, particularly those with dementia, frailty, or at the end of life [171].

## 8. Conclusions

Hypoglycemia remains a major and often under-appreciated challenge to the health, independence and quality of life of older adults living with diabetes. This review proposes a patient- and function-centric approach as a practical and clinically relevant framework for the assessment and management of hypoglycemia, moving beyond glucose targets to address the full spectrum of physical, cognitive, emotional, functional, and social determinants that shape patient outcomes.

By integrating comprehensive functional assessment, individualized pharmacotherapy, emerging glucose monitoring technologies and structured patient and caregiver education into routine care, clinicians can more effectively prevent hypoglycemia and support sustained self-management. Particular attention should be given to atypical and nocturnal presentations of hypoglycemia in the elderly. Medication dosing in the setting of different organ dysfunction such as pancreatic, renal and liver dysfunction should be carefully adjusted. Assessment and management of hypoglycemia unawareness, fear of hypoglycemia, cognitive impairment, frailty, and social vulnerability are essential in this high-risk population.

Recent advances in continuous glucose monitoring (CGM) devices have been shown to improve diabetes management and should be considered a standard of care in older adults with diabetes. Newer medications such as SGLT-2 inhibitors and GLP-1 receptor agonists offer glucose-lowering benefits without the hypoglycemia risk posed by sulfonylureas or insulin. Accessible rescue treatments such as intranasal glucagon provide powerful tools to improve safety and clinical outcomes. When applied within a patient- and function-centered framework, these innovations not only reduce hypoglycemia risk but also preserve functional independence, support emotional well-being, and enhance overall quality of life. However, their use in frail elderly patients requires careful consideration due to a potentially increased risk of adverse effects in this population.Ultimately, adoption of this integrated model of care represents a necessary evolution in diabetes management for the aging population—one that prioritizes safety, dignity, function, and quality of life alongside glycemic control.

## Figures and Tables

**Table 1:** Predisposing and precipitating factors for hypoglycemia. Table adapted from Alagiakrishnan & Mereu, 2010 [11].

Predisposing Factors (Predictors)	Precipitating Factors (Potentiators)
Advanced age History of severe hypoglycemia Insulin use for >10 years Chronic renal failure Chronic liver disease Hypoglycemia unawareness Cognitive impairment HbA1c ≤ 6% (on treatment target)	Certain anti-hyperglycemia agents (insulin, meglitinides, sulfonylureas) Missed or delayed meals Less oral intake than planned Forgetting to eat (especially with dementia) Acute illness Increased physical activity

**Table 2:** Typical and atypical symptoms of hypoglycemia. Adapted from Alagiakrishnan & Mereu, 2010 [11].

Typical Hypoglycemia Symptoms	Atypical Hypoglycemia Symptoms
**Autonomic** Diaphoresis Palpitations Anxiety Hunger Nausea	**Cardiovascular** Myocardial infarction Ventricular arrhythmia Heart failure
**Neuroglycopenic** Headache Dizziness Difficulty concentrating Weakness Slurring of speech Behavioral changes Confusion Somnolence Seizure Coma	**Central nervous system** Vague neurologic symptoms Stroke Cognitive impairment Falls

**Table 3:** Scales to Assess the Burden of Hypoglycemia.

**1.** **Cognitive Burden** **a.** MMSE **b.** MoCA **c.** Mini-Cog **d.** DSM-V Criteria
**2.** **Frailty Burden** **a.** Clinical Frailty Scale **b.** Edmonton Frailty Scale **c.** Frailty Index
**3.** **Clinical Burden** **a.** Hypoglycemia Burden Score **b.** Hypoglycemia Problem Solving Scale (HPSS) **c.** Edinburg Hypoglycemia Scale
**4.** **Psychological Burden** **a.** Fear of Hypoglycemia Questionnaire **b.** Hypoglycemia Fear Survey (HFS) **c.** Problem Areas in Diabetes (PAID) Scale *
**5.** **Social Burden** **a.** Hypoglycemia-related Quality of Life (Hypo-QoL) Scale ** **b.** Diabetes Quality of Life (DQOL) Questionnaire **c.** Diabetes Distress Scale (DDS)

* The PAID scale is a 20-item questionnaire that assesses emotional distress related to diabetes management, including feeling of guilt, frustration, and anxiety. ** The Hypo-QoL scale focuses on how hypoglycemia impacts various aspects of quality of life, including the negative effects on social life and activities.

**Table 4:** HCP Role in Hypoglycemia Assessment.

**1.** Assess the severity of hypoglycemia episodes. **2.** Assess the timing of episodes to determine if the pattern is fasting, postprandial, or nocturnal. **3.** Investigate predisposing and precipitating factors contributing to hypoglycemia. **4.** Ask about hypoglycemia unawareness and fear of hypoglycemia. **5.** Review the medical history and medications that may enhance hypoglycemia risk. **6.** Assess cognitive function, social function, mood, and distress levels in relation to diabetes. **7.** Monitor renal function (creatinine clearance), particularly in elderly patients. **8.** Once risk factors are identified, create a tailored strategy to prevent future episodes. **9.** Set blood glucose goals specific to each patient’s needs and functional status. **10.** Instruct patients on lifestyle modifications and strategies to avoid hypoglycemia, including the effects of alcohol (which can cause delayed hypoglycemia 3–6 h after ingestion) and strenuous physical activity (which may require insulin dose adjustments for up to 24 h). **11.** Select glucose-lowering agents that minimize hypoglycemia risk.

**Table 5:** Adapted from: American Diabetes Association, Glycemic Goals and Hypoglycemia: Standards of Care in Diabetes—2025.

Health Status	HbA1c Target
Healthy (a few comorbidities, intact cognitive and functional status)	<7.0–7.5%
Complex/intermediate (>3 comorbidities, mild-moderate cognitive impairment, >2 IADL impairments)	<8.0%
Long-term care of end-stage chronic diseases, moderate-severe cognitive impairment, >2 IADL impairments	Avoid reliance on HbA1c

## Data Availability

No new data were created or analyzed in this study. Data sharing is not applicable to this article.
